# Revised guidelines for management of steroid-sensitive nephrotic syndrome

**DOI:** 10.4103/0971-4065.41289

**Published:** 2008-01

**Authors:** Arvind Bagga

**Affiliations:** Department of Pediatrics, All India Institute of Medical Sciences, New Delhi, India

**Keywords:** Nephrotic syndrome, practice guidelines, recommendations

## Abstract

**Justification:**

In 2001, the Indian Pediatric Nephrology Group formulated guidelines for management of patients with steroid-sensitive nephrotic syndrome. In view of emerging scientific evidence, it was felt necessary to review the existing recommendations.

**Process:**

Following a preliminary meeting in March 2007, a draft statement was prepared and circulated among pediatric nephrologists in the country to arrive at a consensus on the evaluation and management of these patients.

**Objectives:**

To revise and formulate recommendations for management of steroid-sensitive nephrotic syndrome.

**Recommendations:**

The need for adequate corticosteroid therapy at the initial episode is emphasized. Guidelines regarding the initial evaluation, indications for renal biopsy and referral to a pediatric nephrologist are updated. It is proposed that patients with frequently relapsing nephrotic syndrome should, at the first instance, be treated with longterm, alternate-day prednisolone. The indications for use of alternative immunosuppressive agents, including levamisole, cyclophosphamide, mycophenolate mofetil, and cyclosporin are outlined. The principles of dietary therapy, management of edema, and prevention and management of complications related to nephrotic syndrome are described. These guidelines, formulated on the basis of current best practice, are aimed to familiarize physicians regarding principles of management of children with steroid-sensitive nephrotic syndrome.

## Introduction

Nephrotic syndrome is an important chronic disease in children. About 80% children with idiopathic nephrotic syndrome show remission of proteinuria following treatment with corticosteroids, and are classified as ‘steroid sensitive’. Most patients have multiple relapses, placing them at risk for steroid toxicity, systemic infections, and other complications. A small proportion of patients who are not steroid sensitive (steroid resistant) are also at risk for similar complications and renal insufficiency.

Most pediatricians would encounter patients with nephrotic syndrome in their practice. They should be familiar with management of these patients and be aware of situations in which referral to a pediatric nephrologist is required. Long-term management of these patients should thereafter be a joint effort between the pediatrician and the pediatric nephrologist.

## Objectives

Guidelines on the management of children with nephrotic syndrome were first formulated by the Indian Pediatric Nephrology Group in 2001.^1^ Since a number of studies on management of these patients have been published during the last 7 years, it was felt desirable to review the existing recommendations. Therefore, following a preliminary meeting in New Delhi (7 March 2007), a draft statement was prepared, circulated, and reviewed by pediatric nephrologists across the country (Annexure I). The present document reflects the current opinion on management of patients with steroid-sensitive nephrotic syndrome.

## Recommendations

Important revisions in this document are listed in Table 1.

**Table 1:** Important revisions in this document

Investigations necessary at initial and subsequent evaluation are described. While updating the literature, the Group endorses the existing guidelines on therapy for the initial episode of nephrotic syndrome. The role of other medications, including mycophenolate mofetil, cyclosporine, and tacrolimus in patients with frequent relapses and steroid dependence is discussed and therapeutic choices clarified. Details on dose and duration of therapy with corticosteroids, when coadministered with other agents are included. Guidelines on immunization, isoniazid prophylaxis, and hypertension updated in conformity with recommendations of the Indian Academy of Pediatrics. Management of complications updated.

## Definitions

*Nephrotic syndrome* is characterized by heavy proteinuria, hypoalbuminemia (serum albumin <2.5 g/dl), hyperlipidemia (serum cholesterol >200 mg/dl), and edema.^1^,^2^ *Nephrotic range proteinuria* is present if early morning urine protein is 3+/4+ (on dipstick or boiling test), spot protein/creatinine ratio >2 mg/mg, or urine albumin excretion >40 mg/m^2^ per hour (on a timed-sample). Precise quantitative assessment of proteinuria, including 24-h urine protein measurement is seldom necessary. Definitions for clarifying the course of nephrotic syndrome are shown in Table 2.

**Table 2:** Definitions related to nephrotic syndrome

Remission	Urine albumin nil or trace (or proteinuria <4 mg/m^2^/h) for three consecutive early morning specimens.
Relapse	Urine albumin 3+ or 4+ (or proteinuria >40 mg/m^2^/h) for three consecutive early morning specimens, having been in remission previously.
Frequent relapses	Two or more relapses in initial 6 months or more than three relapses in any 12 months.
Steroid dependence	Two consecutive relapses when on alternate day steroids or within 14 days of its discontinuation.
Steroid resistance	Absence of remission despite therapy with daily prednisolone at a dose of 2 mg/kg per day for 4 weeks.

## Initial Evaluation

A detailed evaluation is necessary before starting treatment with corticosteroids. The height, weight, and blood pressure should be recorded. Regular weight record helps to monitor the decrease or increase of edema. Physical examination is done to detect infections and underlying systemic disorder, e.g., systemic lupus erythematosus, Henoch Schonlein purpura, etc. Infections should be treated before starting therapy with corticosteroids.

Investigations recommended at the initial episode include urinalysis, complete blood count, blood levels of albumin, cholesterol, urea, and creatinine. Estimation of blood levels of antistreptolysin O and C3 is required in patients with gross or persistent microscopic hematuria. Appropriate tests are performed, if necessary, for associated conditions (e.g., chest X-ray and tuberculin test, hepatitis B surface antigen, and antinuclear antibodies). Urine culture is not necessary unless the patient has clinical features suggestive of a urinary tract infection.

## Treatment of the Initial Episode

Adequate treatment of the initial episode, both in terms of dose and duration of corticosteroids, is important. Evidence from multiple studies suggests that appropriate therapy at the first episode of nephrotic syndrome is an important determinant of the long-term course of the disease.^3^

## Medication

The standard medication for treatment is prednisolone or prednisone. The medication is administered after meals to reduce its gastrointestinal side effects. The use of methylprednisolone, dexamethasone, betamethasone, triamcinolone, or hydrocortisone is not recommended. There is also limited evidence on the efficacy or benefits of therapy with deflazocort for nephrotic syndrome.

## Treatment regimen

Various treatment regimens have been used for the treatment of the initial episode of nephrotic syndrome. The International Study for Kidney Diseases in Children had originally recommended a regimen comprising of 4-weeks each of daily and alternate day steroid therapy,^4^ which was used for almost three decades. Controlled studies later suggested that prolongation of initial steroid therapy for 12 weeks or longer is associated with significantly reduced risk for subsequent relapses. However, prolonged treatment with steroids is associated with a higher frequency of adverse events.^3^,^5^,^6^

The Cochrane Renal Group,^3^ on systematic analysis of the literature, recommends that the duration of initial prednisolone therapy should be for a minimum of 12 weeks. It further suggests that the benefits of sustained remission and reduction in relapse rates are superior if alternate-day treatment is not stopped abruptly at 12 weeks, but tapered over the next 2-4 months. It is emphasized that none of the studies included in this analysis was placebo-controlled, most lacked allocation concealment and were not powered to evaluate side effects of prolonged treatment.^3^ The debate regarding appropriate dose and duration of steroid treatment is not resolved. Other regimens are being examined that reduce the risk of relapse without increased side effects.

On the basis of current evidence and opinion, the Group recommends that the initial episode of nephrotic syndrome be treated with prednisolone at a dose of 2 mg/kg per day (maximum 60 mg in single or divided doses) for 6 weeks, followed by 1.5 mg/kg (maximum 40 mg) as a single morning dose on alternate days for the next 6 weeks; therapy is then discontinued. *The benefits and safety of prolonged initial steroid therapy, beyond 12-weeks, require further studies.*

## Treatment of Relapse

The patient should be examined for infections, which should be treated before initiating steroid therapy. Appropriate therapy of an infection might *rarely* result in spontaneous remission, thereby avoiding the need for treatment with corticosteroids.

Prednisolone is administered at a dose of 2 mg/kg/day (single or divided doses) until urine protein is trace or nil for three consecutive days. Subsequently, prednisolone is given in a single morning dose of 1.5 mg/kg on alternate days for 4 weeks, and then discontinued.^1^,^5^ The usual duration of treatment for a relapse is thus 5-6 weeks. Prolongation of therapy is not necessary for patients with infrequent relapses (see below).

In case the patient is not in remission despite 2 weeks treatment with daily prednisolone, the treatment is extended for 2 more weeks. Patients showing no remission despite 4 weeks' treatment with daily prednisolone should be referred for evaluation.

### Infrequent relapsers

Patients who have three or less relapses a year and respond promptly to prednisolone are managed using the aforementioned regimen for each relapse. Such children are at a low risk for developing steroid toxicity.

### Frequent relapsers and steroid dependence

Patients with frequent relapses or steroid dependence should be managed in consultation with a pediatric nephrologist.

It is usually not necessary to perform a renal biopsy in these cases. Following treatment of a relapse, prednisolone is gradually tapered to maintain the patient in remission on alternate day dose of 0.5-0.7 mg/kg, which is administered for 9-18 months. A close monitoring of growth and blood pressure, and evaluation for features of steroid toxicity is essential. If the prednisolone threshold, to maintain remission, is higher than 0.5 mg/kg on alternate days or if features of corticosteroid toxicity are seen, additional use of the following immunomodulators is suggested.

### (a) Levamisole

It is administered at a dose of 2-2.5 mg/kg on alternate days for 12-24 months.^7^–^9^ Cotreatment with prednisolone at a dose of 1.5 mg/kg on alternate days is given for 2-4 weeks; the dose is gradually reduced by 0.15-0.25 mg/kg every 4 weeks to a maintenance dose of 0.25-0.5 mg/kg that is continued for six or more months. Occasionally, it might be possible to discontinue treatment with corticosteroids. The chief side effect of levamisole is leukopenia; flu-like symptoms, liver toxicity, convulsions, and skin rash are rare. The total leukocyte count should be monitored every 12-16 weeks.

### (b) Cyclophosphamide

It is administered at a dose of 2-2.5 mg/kg/day for 12 weeks.^10^ Prednisolone is co-administered at a dose of 1.5 mg/kg on alternate days for 4 weeks, followed by 1 mg/kg for the next 8 weeks; steroid therapy is tapered and stopped over the next 2-3 months. Therapy with cyclophosphamide should be instituted preferably following remission of proteinuria.

Total leukocyte counts are monitored every 2 weeks; treatment with cyclophosphamide is temporarily discontinued if the count falls below 4000/mm^3^. An increased oral fluid intake and frequent voiding prevents the complication of hemorrhagic cystitis; other side effects are alopecia, nausea, and vomiting. The risk of gonadal toxicity is limited with a single (12 weeks) course of cyclophosphamide.^7^,^10^,^11^ The use of more than one course of this agent should preferably be avoided.

In view of its toxicity, the use of chlorambucil, unless under close supervision, is not recommended.

### (c) Calcineurin inhibitors

Cyclosporin (CsA) is given at a dose of 4-5 mg/kg daily for 12-24 months. Prednisolone is coadministered at a dose of 1.5 mg/kg on alternate days for 2-4 weeks; its dose is gradually reduced by 0.15-0.25 mg/kg every 4 weeks to a maintenance dose of 0.25-0.5 mg/kg that is continued for 6 or more months. Occasionally, treatment with corticosteroids may be discontinued.

Estimation of trough blood levels of CsA is required in patients with suspected noncompliance, unsatisfactory response, or nephrotoxicity (increase in serum creatinine by 30% or more from the baseline).^12^ Trough (12-h) CsA levels should be kept between 80 and 120 ng/ml.^12^ Side effects of CsA therapy include hypertension, cosmetic symptoms (gum hypertrophy, hirsutism), and nephrotoxicity; hypercholesterolemia, and elevated transaminases may occur. Estimation of serum creatinine is required every 2-3 months and a lipid profile annually. A repeat kidney biopsy, to examine for histological evidence of nephrotoxicity, should be done if therapy with calcineurin inhibitors is extended beyond 2 years.^12^

Tacrolimus is an alternative agent, administered at a dose of 0.1-0.2 mg/kg daily for 12-24 months. Side effects include hyperglycemia, diarrhea, and rarely neurotoxicity (headache, seizures). The use of tacrolimus is preferred especially in adolescents, because of lack of cosmetic side effects.^13^ Blood levels of creatinine and glucose should be estimated every 2-3 months.

### (d) Mycophenolate mofetil (MMF)

Mycophenolate mofetil is given at a dose of 800-1200 mg/m^2^ along with tapering doses of prednisolone for 12-24 months.^7^,^14^ The principal side effects include gastrointestinal discomfort, diarrhea, and leukopenia. Leukocyte counts should be monitored every 1-2 months; treatment is withheld if count falls below 4000/mm^3^.

#### Choice of agent

The advantages of using these drugs should be balanced against their potential toxicity. There are few studies comparing one agent with another, but evidence for efficacy is strongest for cyclophosphamide and CsA. Levamisole has a modest steroid sparing effect and is a satisfactory initial choice for patients with frequent relapses or steroid dependence. Treatment with cyclophosphamide is preferred in patients showing: (i) significant steroid toxicity, (ii) severe relapses with episodes of hypovolemia or thrombosis, and (iii) poor compliance or difficult follow-up, where 12 weeks therapy might be possible to ensure than long-term compliance.

Treatment with CsA or tacrolimus is recommended for patients who continue to show steroid dependence or frequent relapses despite treatment with the above medications.^12^ Either of these agents is effective in maintaining remission in most patients with steroid-sensitive nephrotic syndrome. The chief concern with their use is nephrotoxicity, but with careful assessment of renal function, minimizing the maintenance dose and utilizing renal biopsies in those receiving prolonged therapy, this risk can be minimized. Recent case series^14^ and a controlled trial^15^ support the use of MMF as a steroid sparing agent. *The lack of renal, hemodynamic and metabolic toxicity with this agent makes it an attractive alternative to calcineurin inhibitors.*

In some patients receiving therapy with levamisole, MMF and calcineurin inhibitors, treatment with prednisolone might be tapered and discontinued after 6-12 months. Some patients who respond to therapy with levamisole, MMF, and calcineurin inhibitors may relapse once these medications are discontinued. Relapses during or following therapy with these agents are treated with prednisolone as described above.

#### Failure of alternative medication

**Fig. 1:**
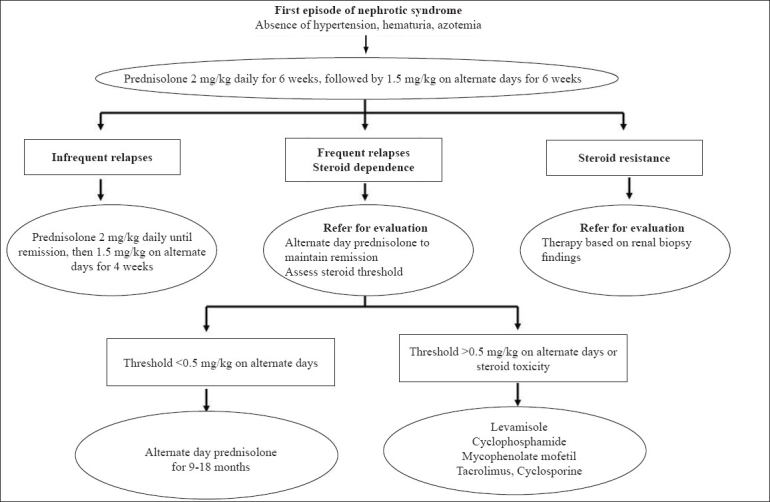
Management of patients with steroid-sensitive nephrotic syndrome

If a patient has two or more relapses over 6 months while on treatment with any of the above agents, its replacement with an alternative medication should be considered. A protocol summarizing the management of patients with steroid-sensitive nephrotic syndrome is shown in Fig. 1.

## Supportive Care

This forms an important aspect of managing children with nephrotic syndrome.

### Diet

A balanced diet, adequate in protein (1.5-2 g/kg) and calories is recommended. Patients with persistent proteinuria should receive 2-2.5 g/kg of protein daily.^16^ Not more than 30% calories should be derived from fat and saturated fats avoided. While salt restriction is not necessary in most patients with steroid-sensitive nephrotic syndrome, reduction of salt intake (1-2 g per day) is advised for those with persistent edema. Salt should not be added to salads and fruits, and snacks containing high salt should be avoided. Since treatment with corticosteroids stimulates appetite, parents should be advised regarding ensuring physical activity and preventing excessive weight gain.

### Edema

Control of edema is an integral part of supportive care. Since treatment with corticosteroids usually leads to diuresis within 5-10 days, diuretics are avoided unless edema is significant. Diuretics should also not be given to patients with diarrhea, vomiting, or hypovolemia.

Patients with persistent edema and weight gain of 7-10% are treated with oral frusemide (1-3 mg/kg daily). Additional treatment with potassium sparing diuretics is not required if frusemide is used at this dose for less than 1 week. Patients requiring higher doses and prolonged duration of treatment with frusemide should receive potassium-sparing diuretics, e.g., spironolactone (2-4 mg/kg daily). Blood pressure should be monitored frequently. A gradual reduction of edema, over 1 week, is preferred.^16^,^17^

**Fig. 2:**
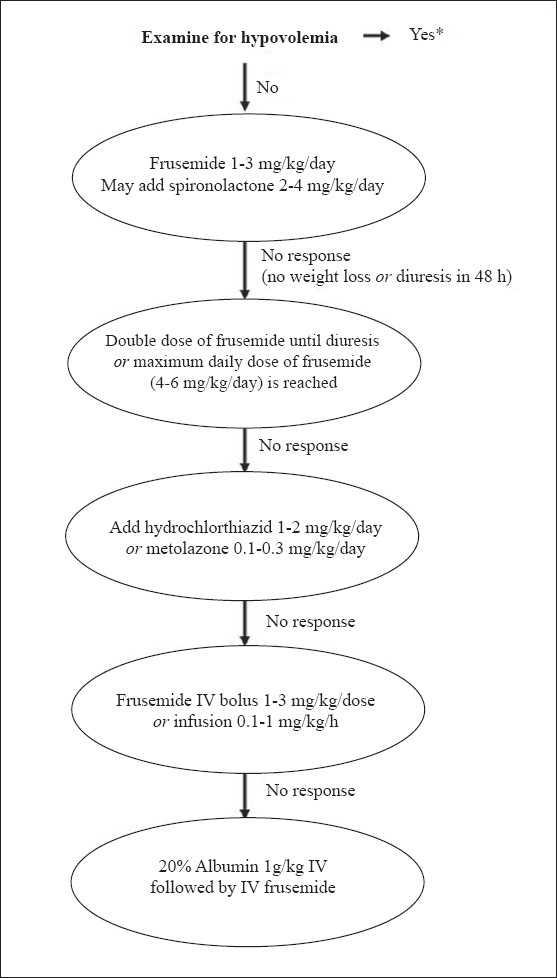
Management of edema in patients with nephrotic syndrome. Patients requiring high-dose frusemide or addition of other diuretics should be under close supervision, preferably in a hospital. Monitoring of serum electrolytes is necessary in all patients receiving diuretics. Patients showing hypokalemia require potassium supplements or coadministration of spironolactone. The medications are reduced stepwise once diuresis ensues. *Management of hypovolemia consists of rapid infusion of normal saline at a dose of 15-20 ml/kg over 20-30 min; this may be repeated if clinical features of hypovolemia persist. Infusion of 5% albumin (10-15 ml/kg) or 20% albumin (0.5-1 g/kg) may be used in subjects who do not respond despite two boluses of saline

Edema not responding to the above therapy should be managed in a hospital. A combination of a loop and thiazide diuretic, and/or a potassium-sparing agent is occasionally necessary. For patients with refractory edema, a combination of diuretics and albumin infusion is used. Albumin (20%) is given as an infusion at a dose of 0.5-1 g/kg over 2-4 h, followed by administration of frusemide (1-2 mg/kg intravenously). While infusion of albumin results in increased urine output, the effect is not sustained and repeated administration might be necessary. Albumin should be administered very cautiously in patients with renal failure, pneumonia or pulmonary edema due to its potential to increase the plasma volume. Patients receiving albumin should be observed for respiratory distress, hypertension, and congestive heart failure. Refractory ascites interfering with respiration or associated with breaks in the skin may be removed by cautious paracentesis. A protocol for treatment of edema is shown in Fig. 2.

### Patient and parent education

Long-term outcome of children with steroid-sensitive nephrotic syndrome is satisfactory, with the majority in sustained remission and with normal renal functions by adolescence. A proportion of patients, especially those (i) with early onset of nephrotic syndrome, (ii) with a frequently relapsing course, and (iii) requiring treatment with alkylating agents or CsA may continue to show relapses beyond adolescence.^18^ Parents should be reassured that despite a relapsing course, progression to end-stage renal failure necessitating dialysis or transplantation is extremely rare.

Parental motivation and involvement is essential in the long-term management of these children. They should be provided information about the disease, its expected course and risk of complications. The following are emphasized:

Urine examination for protein can be done at home using dipstick, sulfosalicylic acid, or boiling test. The examination should be done every morning during a relapse, during intercurrent infections or if there is even mild periorbital puffiness. The frequency of urine examination is reduced, to once or twice a week, during remission. The importance of detecting relapse before development of significant edema is stressed.

Patients should be advised to maintain a diary showing results of urine protein examination, medications received, and intercurrent infections.

Normal activity and school attendance should be ensured; the child should continue to participate in all activities and sports.

Since infections are an important cause of morbidity, patients should receive appropriate immunization and other measures for protection.

## Other Medications

The use of antacids or histamine receptor antagonists (e.g., ranitidine) is not necessary, unless there are symptoms of upper gastrointestinal discomfort. Long-term calcium supplementation (calcium carbonate, 250-500 mg) is necessary if the patient receives more than 3 months treatment with prednisolone.^19^ Patients with steroid-sensitive nephrotic syndrome do not usually require medications for hyperlipidemia, since lipids normalize following remission.

## Immunization

Parents should be advised regarding the need for completing the primary immunization. Administration of some vaccines, e.g., hepatitis B, measles-mumps-rubella or meningococcal vaccines may rarely precipitate a relapse.

Patients receiving prednisolone at a dose of 2 mg/kg/day or greater, or total 20 mg/day or greater (for patients weighing >10 kg) for more than 14 days are considered immunocompromized.^20^ Such patients should not receive live attenuated vaccines; inactivated or killed vaccines are safe.^20^ Live vaccines are administered once the child is off immunosuppressive medications for at least 4 weeks. If there is a pressing need, these vaccines may be given to patients receiving alternate day prednisolone at a dose <0.5 mg/kg.

All children with nephrotic syndrome should receive immunization against pneumococcal infections.^21^ It is important to note that not all pneumococcal serotypes are included in the vaccines and that antibody levels may decline during a relapse. Previously vaccinated children may, therefore, develop pneumococcal peritonitis and sepsis. The Expert Group endorses the recommendations of the Immunization Committee of the Indian Academy of Pediatrics.^22^ The Committee recommends 2-4 doses of the heptavalent conjugate pneumococcal vaccine for children below 2 years of age. For previously unimmunized children between 2 and 5 year old, a priming dose of the conjugate vaccine should be followed 8 weeks later, by a dose of the 23-valent polysaccharide vaccine. Children older than 5 years require only a single dose of the polysaccharide vaccine. The vaccine should be given during remission, preferably when the child is not receiving daily prednisolone. Revaccination after 5 years is considered for children (<10-year-old) with active nephrotic syndrome.

Patients in remission and not on immunosuppressive therapy should receive the varicella vaccine. One dose is recommended for children between 12 months and 12 years of age, and two doses separated by an interval of at least 4 weeks for children 13 years or older.^23^

## Kidney Biopsy

Children with idiopathic nephrotic syndrome not having hematuria, hypertension, or impaired renal function are treated with corticosteroids without requiring a kidney biopsy. A biopsy is usually not necessary in patients with frequent relapses or steroid dependence before starting treatment with levamisole, cyclophosphamide, or MMF, but should be performed before therapy with calcineurin inhibitors. A biopsy is required to identify the underlying renal disease in certain cases [Table 3].

**Table 3:** Indications for kidney biopsy

At onset
Age of onset <1 year.
Gross hematuria, persistent microscopic hematuria or low serum C3.
Sustained hypertension.
Renal failure not attributable to hypovolemia.
Suspected secondary causes of nephrotic syndrome.
**After initial treatment**
Proteinuria persisting despite 4-weeks of daily corticosteroid therapy.
Before treatment with cyclosporin A or tacrolimus.

Kidney biopsies must be performed by experts with experience in the procedure. Centers that perform kidney biopsies should have facilities for evaluation of the specimens by light and immunofluorescence microscopy.

## Referral to Pediatric Nephrologist

Indications for referral of patients are given in Table 4. The care of these patients should be a joint collaboration between the pediatrician and pediatric nephrologist.

**Table 4:** Indications for referral to a pediatric nephrologist

Onset below 1-year of age; family history of nephrotic syndrome. Nephrotic syndrome with hypertension, gross/persistent microscopic hematuria, impaired renal function, or extrarenal features (e.g., arthritis, serositis, and rash). Complications: refractory edema, thrombosis, severe infections, and steroid toxicity. Resistance to steroid therapy. Frequently relapsing or steroid dependent nephrotic syndrome.

## Complications

Patients with steroid-sensitive nephrotic syndrome are at risk for certain complications, early detection of which is necessary.

### Infections

Children with nephrotic syndrome are susceptible to severe infections, which need prompt treatment. Common infections include peritonitis, cellulites, and pneumonia. Viral and bacterial infections may occasionally precipitate relapses in patients previously in remission. The clinical features and management of common serious infections are summarized in Table 5.

**Table 5:** Clinical features and management of infections*

Infection	Clinical features	Common organisms	Antibiotics, duration of treatment
Peritonitis	Abdominal pain, tenderness, distension; diarrhea, vomiting; ascitic fluid >100 leukocytes/mm^3^; >50% neutrophils	*S. pneumoniae, S. pyogenes, E. coli*	Cefotaxime or ceftriaxone for 7-10 days; ampicillin and an aminoglycoside for 7-10 days
Pneumonia	Fever, cough, tachypnea, intercostal recessions, crepitations	*S. pneumoniae, H. influenzae, S. aureus*	*Oral*: amoxicillin, co-amoxiclav, erythromycin
			*Parenteral*: ampicillin and aminoglycoside; or cefotaxime/ceftriaxone for 7-10 days
Cellulitis	Cutaneous erythema, induration, tenderness	Staphylococci, Group A streptococci, *H. influenzae*	Cloxacillin and ceftriaxone for 7-10 days co-amoxiclav
Fungal infections	Pulmonary infiltrates, persistent	*Candida, Aspergillus* spp.	Skin, mucosa: fluconazole for 10 days
	Fever unresponsive to antibiotics, sputum/urine showing septate hyphae		Systemic: amphotericin for 14-21 days

* Supplemental stress doses of hydrocortisone or prednisolone are usually necessary

Varicella may be a severe illness in patients with nephrotic syndrome receiving corticosteroids or other immunosuppressive drugs. Susceptible patients (those unimmunized or with no history of varicella) who are exposed to a case of chickenpox should, therefore, receive a single dose of varicella zoster immunoglobulin, within 96 h of exposure to prevent or lessen the severity of the disease.^17^ Since, this preparation is expensive and not easily available, a single dose of intravenous immunoglobulin (400 mg/kg) may be used instead.^23^ However, no clinical data showing the effectiveness of the latter strategy are available.

Patients who develop varicella should receive intravenous acyclovir (1500 mg/m^2^/day in three doses) or oral acyclovir (80 mg/kg/day in four doses) for 7-10 days.^23^ The dose of prednisolone should be tapered to 0.5 mg/kg/day or lower during the infection.

Patients with nephrotic syndrome who are Mantoux positive, but show no evidence of tuberculosis should receive prophylaxis with isoniazid (INH) for 6 months.^24^ Those showing evidence of active tuberculosis should receive standard therapy with antitubercular drugs.

### Thrombosis

Children with nephrotic syndrome are at risk for venous and, rarely, arterial thrombosis.^16^,^17^ Reduced intravascular volume, immobilization, indwelling vascular catheters, aggressive diuretic use, and puncture of deep vessels predispose to thrombus formation. Renal vein thrombosis is suspected in a patient with oligoanuria, hematuria, or flank pain, especially following an episode of dehydration. Femoral and mesenteric arterial thrombosis may occasionally occur. Deep vein thrombosis of calf veins is less common in children, but may lead to pulmonary embolism. Saggital sinus and cortical venous thrombosis may follow episodes of diarrhea and present with convulsions, vomiting, altered sensorium, and neurological deficits. Ultrasonography, Doppler studies, and cranial magnetic resonance imaging (MRI) are useful in confirming the diagnosis.

Patients with thrombotic complications require urgent treatment. The treatment includes correction of dehydration and other complications, and use of heparin (IV) or low-molecular-weight heparin (subcutaneously) initially, followed by oral anticoagulants on the longterm.^16^,^17^ There is no role for prophylactic treatment with anticoagulants in patients with hypoalbuminemia and edema.

### Hypertension

This may be detected at the onset of nephrotic syndrome or later due to steroid toxicity. Therapy is initiated with angiotensin converting enzyme (ACE) inhibitors, calcium channel blockers or b adrenergic antagonists, keeping the blood pressure at less than the 90th percentile.^25^

### Hypovolemic shock

This complication can occur due to unsupervised use of diuretics especially if accompanied by septicemia, diarrhea, or vomiting. The diagnosis is suggested by moderate to severe abdominal pain, hypotension, tachycardia, cold extremities, and poor capillary refill; hematocrit and blood levels of urea, and uric acid are elevated. Management consists of rapid infusion of normal saline at a dose of 15-20 ml/kg over 20-30 min; this is repeated if clinical features of hypovolemia persist. Infusion of 5% albumin (10-15 ml/kg) or 20% albumin (0.5-1 g/kg) may be used in subjects who do not respond despite two boluses of saline.

### Corticosteroid side effects

Prolonged steroid therapy may be associated with significant side effects. Patients (if they can understand) and the parents should be explained about the side effects of the medications, including increased appetite, impaired growth, behavioral changes, risk of infections, salt and water retention, hypertension, and bone demineralization. All patients should be monitored for cushingoid features and blood pressure; six-monthly record of height and weight, and yearly evaluation for cataract should be done. Patients on prolonged (>3 months) treatment with steroids should receive daily supplements of oral calcium (250-500 mg daily) and vitamin D (125-250 IU).^19^

### Steroids during stress

Patients who have received high-dose steroids for more than 2 weeks in the past year are at risk of suppression of the hypothalamo-pituitary-adrenal axis. These children require supplementation of steroids during surgery, anesthesia, or serious infections.^26^ Corticosteroids are supplemented, as parenteral hydrocortisone at a dose of 2-4 mg/kg/day, followed by oral prednisolone at 0.3-1 mg/kg/day. This is given for the duration of stress and then tapered rapidly.

## Conclusions

Recommendations on management of nephrotic syndrome, proposed in 2001, have been reexamined and revised based on systematic reviews, published studies, and expert opinion of the members of the Indian Pediatric Nephrology Group. These guidelines are intended to familiarize physicians with principles of management of children with steroid-sensitive nephrotic syndrome. Therapy needs to be individualized for each patient and optimal care will be achieved by combined inputs of the primary pediatrician and pediatric nephrologist. Further revisions of these guidelines, indicating best current practice, shall be periodically necessary.

## Annexure I: Members of the Review Committee

Kamran Afzal, Jawaharlal Nehru Medical College, Aligarh; Indira Agarwal, Christian Medical College Hospital, Vellore; Vinay Agarwal, Max Hospital, New Delhi; Uma Ali, Bai Jerbai Wadia Hospital for Children, Mumbai; Sanjeev Bagai, Rockland Hospital, New Delhi; Arvind Bagga, All India Institute of Medical Sciences, New Delhi (*Convenor*); Sushmita Banerjee, Calcutta Medical Research Institute, Kolkata; Ashima Gulati, All India Institute of Medical Sciences, Delhi; Sanjeev Gulati, Fortis Hospital, New Delhi; Pankaj Hari, All India Institute of Medical Sciences, New Delhi (*Secretary*); Arpana Iyengar, St. John's Medical College, Bangalore; OP Jaiswal, Sunder Lal Jain Hospital, New Delhi; Rupesh Jain, Ekta Hospital for Children, Raipur; M Kanitkar, Armed Forces Medical College, Pune; Mukta Mantan, Maulana Azad Medical College, New Delhi; Kamini Mehta, Lilavati Hospital and Research Center, Mumbai; Kumud Mehta, Jaslok Hospital and Research Center and Bai Jerbai Wadia Hospital for Children, Mumbai; BR Nammalwar, Kanchi Kamakoti CHILDS Trust Hospital, Chennai; Amitava Pahari, Apollo Hospital, Kolkata; Saroj K Patnaik, No. 12 Air Force Hospital, Gorakhpur; KD Phadke, St. John's Medical College, Bangalore; PK Pruthi, Sir Gangaram Hospital, New Delhi; Abhijeet Saha, Government Medical College, Chandigarh; VK Sairam, Sri Ramchandra Medical College, Chennai; Jayati Sengupta, AMRI Hospital, Kolkata; Prabha Senguttuvan, Institute of Child Health, Chennai (*Chairperson*); Sidharth K Sethi, All India Institute of Medical Sciences, New Delhi; Mehul Shah, Apollo Hospital, Hyderabad; Jyoti Sharma, Bharti Vidyapeeth Medical College, Poona; RN Srivastava, Indraprastha Apollo Hospital, New Delhi; AS Vasudev, Indraprastha Apollo Hospital, New Delhi; Anil Vasudevan, St. John's Medical College, Bangalore; and M Vijayakumar, Mehta Children's Hospital, Chennai.
